# A novel enema formulation containing cannabidiol and sodium propionate for ulcerative colitis: preclinical assessment in a murine model

**DOI:** 10.1186/s42238-026-00400-4

**Published:** 2026-03-24

**Authors:** Timna Naftali, Sari Prutchi Sagiv

**Affiliations:** 1 Meir Medical Center, Kfar Saba, Israel; 2 CannaMore Biotechs Ltd, Bnei Brak, Israel; 3 Gray Faculty of Medicine and Health Sciences, Tel Aviv University, Tel Aviv, Israel

**Keywords:** Ulcerative colitis, Cannabidiol, Cannabis, Sodium propionate, Enema formulation, Inflammatory bowel disease, DSS-induced colitis, Short-chain fatty acids

## Abstract

**Background:**

Ulcerative colitis (UC) is a chronic inflammatory bowel disease characterized by mucosal inflammation, epithelial barrier disruption, and immune dysregulation. Cannabidiol (CBD), a non-psychoactive phytocannabinoid, and sodium propionate (Na-P), a short-chain fatty acid, posses complementary anti-inflammatory and epithelial-protective properties. This study aimed to evaluate the safety and efficacy of a novel rectal enema formulation combining CBD and Na-P in a murine model of dextran sodium sulfate (DSS)-induced colitis.

**Methods:**

Experimental colitis was induced in C57BL/6 mice via 2.5% DSS administration for five days. Mice received one of 4 daily rectal treatments from day 4 to day 11: saline, hyaluronic acid (HA, vehicle), HA + CBD, HA + Na-P, or HA + CBD+Na-P. Clinical disease activity was monitored through the Disease Activity Index (DAI), body weight, colon length, and histopathological evaluation.

**Results:**

Colon length analysis demonstrated a 34.7% increase in length in the combination group versus saline control, significantly exceeding improvements observed with CBD or Na-P alone (*p* < 0.001). The combination treatment of CBD and Na-P led to a significant and sustained reduction in DAI scores compared to controls and monotherapies. Notably, the combination group exhibited a steeper improvement in DAI from day 6 onwards and showed superior outcomes on day 11 (*p* < 0.004 vs. HA). Body weight loss was markedly attenuated in the combination group, with statistical significance observed from day 6 (*p* < 0.05), while single-agent treatments exhibited only partial benefit. Histopathological scores were reduced in the combination group, accompanied by decreased neutrophilic infiltration and attenuation of mucosal damage, though statistical significance was not reached compared to monotherapies. Importantly, no hemorrhages or fibrin deposits were observed in any group, supporting the safety profile of the combination treatment.

**Conclusions:**

This preclinical study demonstrates that rectal administration of a CBD and Na-P enema exerts potential synergistic therapeutic effects in experimental UC, effectively reducing disease severity, preserving colon morphology, and mitigating histopathological damage. These findings support further clinical investigation of this novel dual-targeted approach for UC management.

## Background

Ulcerative colitis (UC) is a chronic, debilitating inflammatory bowel disease (IBD) characterized by recurrent inflammation of the colonic mucosa, leading to symptoms such as abdominal pain, persistent diarrhea, rectal bleeding, and mucosal ulceration (Ungaro et al. 2017). The disease typically originates in the rectum and extends proximally in a continuous manner, affecting variable lengths of the colon. UC follows a relapsing-remitting course and is associated with a substantial reduction in patients’ quality of life.

Although the precise etiology of UC remains incompletely understood, it is widely regarded as a multifactorial disorder involving genetic susceptibility, immune dysregulation, environmental factors, and alterations in the gut microbiota (Ananthakrishnan 2015). Persistent immune activation is driven by pro-inflammatory cytokines, including tumor necrosis factor-α (TNF-α), interleukin-1β (IL-1β), and interleukin-6 (IL-6), which contribute to sustained mucosal inflammation and tissue damage (Ahluwalia et al. 2018). In parallel, impairment of the mucosal barrier and loss of immune tolerance further amplify the inflammatory cascade, promoting disease chronicity and increasing symptom severity (Neurath 2019).

Current therapeutic strategies for UC aim to reduce inflammation, achieve clinical remission, and maintain long-term mucosal healing. Aminosalicylates are the cornerstone of treatment for mild to moderate disease, while corticosteroids are commonly used to control acute flares (Ko et al. 2019). In patients with moderate to severe UC, immunosuppressive agents (e.g., azathioprine) and biologic therapies targeting key inflammatory pathways, such as anti-tumor necrosis factor (TNF) agents and interleukin inhibitors, are frequently employed (Ungaro et al. 2017). The extent of colonic involvement varies among patients, ranging from pancolitis, in which the entire colon is affected, to more limited disease involving shorter segments, including isolated rectal involvement (proctitis). Despite the seemingly limited extent of disease, proctitis can be severe and significantly impair patients’ quality of life. Initial treatment is typically local, using 5-aminosalicylic acid (5-ASA) and corticosteroids administered as enemas or suppositories; however, these therapies often fail, necessitating escalation to systemic immunosuppressive or biologic treatments.

In addition, current therapies are associated with adverse effects, risk of relapse, and incomplete response rates. Consequently, there remains a pressing need for novel, targeted therapeutic approaches with improved safety profiles and sustained long-term efficacy.

Cannabidiol (CBD), a non-psychoactive constituent of *Cannabis sativa*, has emerged as a promising therapeutic agent due to its immunomodulatory, anti-inflammatory, and antioxidant properties. Unlike Δ9-tetrahydrocannabinol (THC), CBD does not produce psychotropic effects, making it an attractive candidate for therapeutic applications (Atalay et al. 2020). Mechanistically, CBD exerts its effects through interactions with the endocannabinoid system, modulating cannabinoid receptors (CB1 and CB2) as well as other molecular targets, including transient receptor potential vanilloid type 1 (TRPV1) channels and peroxisome proliferator-activated receptors (PPARs) (Bilkei-Gorzo et al. 2023). Preclinical studies have demonstrated that CBD suppresses the production of pro-inflammatory cytokines, such as TNF-α and IL-6, while promoting regulatory T-cell activity (Malfait et al. 2000).

Clinical studies have further highlighted the therapeutic potential of CBD in the management of IBD. We have previously demonstrated that administration of CBD-rich cannabis improved clinical outcomes, reduced abdominal pain, and enhanced quality of life in patients with UC (Naftali et al. 2021). Similarly, Irving et al. (Irving et al. 2018) and Borrelli et al. (Borrelli et al. 2009) reported reductions in inflammatory markers and improvements in histological outcomes in UC models and patients treated with cannabinoids, supporting a potential role for CBD in modulating disease activity and promoting mucosal healing. Importantly, CBD has shown a favorable safety profile across various routes of administration, even at relatively high doses, with no severe adverse effects reported in clinical trials, further supporting its suitability for chronic inflammatory conditions such as IBD (Bergamaschi et al. 2011).

Short-chain fatty acids (SCFAs), including acetate, butyrate, and propionate, are key metabolites generated by the fermentation of dietary fiber by the gut microbiota. SCFAs play an essential role in maintaining gut homeostasis by modulating immune responses, enhancing epithelial barrier integrity, and reducing oxidative stress (Morrison and Preston 2016). Propionate, in particular, has been shown to activate G-protein–coupled receptors (GPR41 and GPR43) on colonic epithelial and immune cells, thereby exerting anti-inflammatory and immunoregulatory effects. These effects include inhibition of nuclear factor-κB (NF-κB) signaling, enhanced differentiation of regulatory T cells, and improved mucosal barrier function (Tan et al. 2014). As a naturally occurring microbial metabolite, propionate is well tolerated and has demonstrated a favorable safety profile in various therapeutic applications, including intrarectal administration, making it an attractive candidate for localized treatment of colonic inflammation (Hosseini et al. 2011).

Given the central role of SCFAs in UC pathogenesis, sodium propionate (Na-P) has gained increasing attention as a potential therapeutic agent capable of counteracting dysregulated immune responses and epithelial dysfunction in UC.

The complementary mechanisms of action of CBD and Na-P suggest potential synergistic benefits when administered together. CBD’s capacity to modulate immune responses and suppress pro-inflammatory cytokine production, combined with propionate’s role in strengthening epithelial barrier integrity and promoting regulatory T-cell differentiation, provides a multifaceted therapeutic strategy for UC. By simultaneously targeting key pathogenic processes - including inflammation, immune dysregulation, and barrier dysfunction - this combination may offer superior therapeutic benefit compared with monotherapy approaches.

In this study, we aimed to evaluate the safety and efficacy of a novel enema formulation containing CBD and Na-P in a dextran sodium sulfate (DSS)–induced mouse model of UC. The DSS model is well established and recapitulates several key features of human UC, including colonic inflammation, epithelial injury, and immune cell infiltration (Chassaing et al. 2014). Our investigation included comprehensive assessments of colon length, clinical disease activity and histopathological changes. By examining the potential synergistic effects of this combination therapy, we sought to provide insight into its therapeutic potential and underlying mechanisms of action in the management of UC.

## Methods and materials

### Induction of DSS colitis

A total of 30 female C57BL/6 mice were used in this study. Animals were obtained at 7–8 weeks of age, with an initial body weight ranging from 19 to 25 g. An acclimatization period of 11 days was provided to allow stabilization and attainment of an average body weight of approximately 22 g prior to study initiation.

Experimental colitis was induced by administration of 2.5% dextran sulfate sodium (DSS) dissolved in drinking water, provided ad libitum for five consecutive days (Day 0–5). The DSS solution was freshly prepared and replaced daily to ensure stability and consistent exposure throughout the induction period. Rectal administration of the test formulations was initiated on Day 4, following three days of DSS exposure.

After DSS induction and prior to the initiation of treatment, animals were randomly assigned to experimental groups using a stratified randomization sequence to ensure comparable mean body weights (± 5%) across groups. The individual animal was considered the experimental unit. Clinical assessment of the Disease Activity Index (DAI) and colon length was performed by a single technician blinded to treatment allocation throughout the study. Administration of the test formulations was carried out by a separate technician to maintain blinding. Histopathological evaluations were conducted by an independent, board-certified pathologist (Pathologica Ltd.) who was also blinded to treatment assignment. All animals were monitored daily for general health, behavior, and clinical signs of distress throughout the experimental period.

### Animal management

#### Housing

All animal handling procedures were conducted in accordance with the guidelines of the National Institutes of Health (NIH) and the Association for Assessment and Accreditation of Laboratory Animal Care (AAALAC). Mice were housed in standard polyethylene cages (six animals per cage; 35 × 30 × 15 cm) equipped with stainless-steel wire lids and provided with ad libitum access to pelleted chow and water. Bedding consisted of steam-sterilized paddy husk (Harlan Sani-Chip, Cat. No. 7090 A) and was replaced at least twice weekly in conjunction with routine cage cleaning.

#### Diet

Animals were provided ad libitum access to a commercial rodent diet (Harlan Teklad TRM Rat/Mouse Diet, Cat. No. 2018SC), which was sterilized prior to use. Drinking water was acidified (pH 2.5–3.5) and autoclaved before provision. Both food and water were available continuously throughout the study.

#### Contaminant control

All feed batches were accompanied by vendor-issued certificates of analysis confirming the absence of contaminants. The water supply was treated as described above to ensure sterility and consistency throughout the experimental period.

#### Environmental conditions

Animals were maintained in individually ventilated cages (IVCs) within a dedicated, temperature- and humidity-controlled facility (22 ± 2 °C; relative humidity 55 ± 15%). Environmental parameters were continuously monitored. The animal room was isolated from external light and maintained on an automated 12-hour light/dark cycle.

#### Facility

All experiments were conducted at SIA, a contract research organization (CRO) specializing in preclinical research, located in the Weizmann Science Park, Ness Ziona, Israel. SIA is certified to conduct animal studies by the Israeli Ministry of Health Animal Care and Use National Committee. The animal facility operates under continuous veterinary supervision, overseen by Dr. Alon Yaar (license number 1284) and the national committee for animal care and use.

### Drugs and treatment administration

On Day 4, mice were randomly assigned, as described above, to one of the following treatment groups, with six animals per group: (1) saline control; (2) vehicle control consisting of hyaluronic acid (HA; 100 µL); (3) HA (100 µL) supplemented with cannabidiol (CBD; 15 mg/kg); (4) HA supplemented with sodium propionate (Na-P; 75 mg/kg); and (5) HA supplemented with the combination of CBD (15 mg/kg) and Na-P (75 mg/kg).

The doses of CBD and Na-P were selected based on translational relevance and prior clinical experience. CBD was administered at 15 mg/kg, corresponding to a mouse-equivalent dose derived from previous personal clinical experience in which oral CBD doses of approximately 300 mg/day were used in patients with Crohn’s disease and graft-versus-host disease (Yeshurun et al. 2015). For the present study, a lower human-equivalent dose of ~ 200 mg was intentionally selected, reflecting the intrarectal route of administration and its expected higher local bioavailability, and was converted to the murine dose using standard body-surface-area scaling (Reagan-Shaw et al. 2008). Na-P was administered at 75 mg/kg based on published preclinical studies demonstrating anti-inflammatory and gut-barrier–protective effects at comparable doses in murine models of intestinal inflammation(Fachi et al. 2020).

**Fig. 1:**
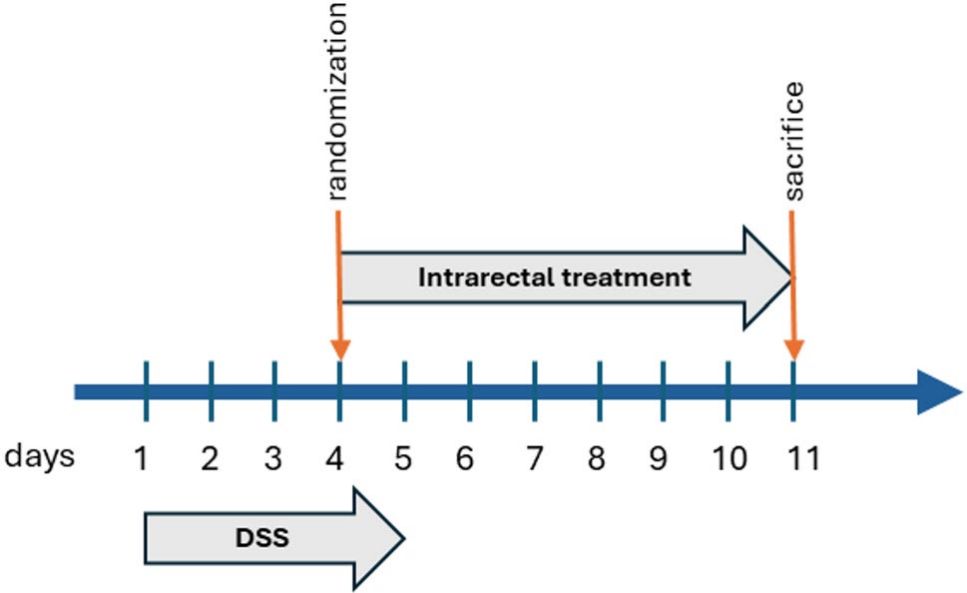
Experimental timeline and treatment regimen. Colitis was induced by 2.5% DSS in drinking water (Day 0–5). Rectal treatments were administered daily from Day 4 to Day 11. Animals were randomized to receive saline, HA, CBD, Na-P, or CBD + Na-P. Animals were sacrificed on Day 11

For intrarectal administration, mice were anesthetized with isoflurane delivered via a gas anesthesia system (2% isoflurane in oxygen at a flow rate of 3 L/min). Once adequate anesthesia was achieved, the designated treatment formulation was administered rectally, approximately 2.5 cm into the distal colon, using a 24-gauge flexible polyethylene catheter. Following administration, the catheter was gently withdrawn, and each mouse was held in a head-down position with the tail elevated for approximately 20 seconds to facilitate retention of the formulation before being returned to its cage for recovery. The treatments were administered daily until animal sacrifice on Day 11 (D11) (see Fig. 1 for the schematic representation of the treatment regimen).

### Disease activity index (DAI)

Throughout the study, animals were monitored daily for abnormal behavior and clinical signs of colitis, including weight loss, bleeding, and stool consistency.

The daily DAI was calculated using a 0 to 4 scoring system based on the following parameters: change in weight (0, ≤ 1%; 1, 1–5%; 2, 5–10%; 3, 10–15%; and 4, > 15%), intestinal bleeding (0, negative; 4, positive), and stool consistency (0, normal; 2, loose stools; 4, diarrhea).

The combined scores were averaged to yield the final daily DAI for each animal.

### Colon length

On Day 11, following euthanasia, the entire colon was excised from each animal and assessed for length and weight. Gross pathological evaluations were performed to document colon size, wall thickness, and the presence of edema. Each colon was photographed with appropriate labeling prior to further processing.

### Colon histological evaluation

Following assessment of colon length and macroscopic damage, segments of the distal colon were stapled flat, mucosal side up, onto cardboard and fixed in 10% neutral-buffered formalin for 24 hours. After fixation, tissues were dehydrated, embedded in paraffin, and sectioned at a thickness of 5 μm. Standard hematoxylin and eosin (H&E) staining was performed for histological evaluation.

Histopathological severity was assessed using a semi-quantitative scoring system. The following parameters were evaluated at low magnification (×4 and ×10): inflammation (0–2), inflammatory cell infiltration (0–2), erosions (0–3), and crypt loss (0–2). The total histopathological score for each section was calculated as the sum of individual parameters, yielding a maximum possible score of 9, in accordance with the scoring scheme described by Shah et al. (Shah et al. 2011). To further characterize tissue healing and inflammatory features, additional histopathological parameters were evaluated at higher magnification (×20), including inflammatory cellular response, lymphocyte infiltration, erosions, crypt loss, inflammatory cell differentiation, hemorrhages, fibrin deposition, lesion distribution, and qualitative description of lesion distribution, as previously described (Erben et al. 2014).

### Study termination

On Day 11 following DSS induction, all animals were euthanized by carbon dioxide (CO₂) inhalation in accordance with institutional animal welfare guidelines.

### Ethical considerations

The study protocol was reviewed and approved by the Meir Medical Center Institutional Ethics Committee on August 8, 2021. The study was conducted in accordance with the principles of the Declaration of Helsinki and Good Clinical Practice guidelines. Ethics approval reference number: NPC-Sc-IL-2108-121-4.

### Study endpoints

The primary endpoint of the study was colon length measured at Day 11, a validated macroscopic indicator of disease severity in DSS-induced colitis (Peyrin-Biroulet et al. 2015). The primary comparison assessed whether treatment with the combined treatment enema resulted in greater preservation of colon length relative to control (saline and HA vehicle) and monotherapy (CBD or Na-P alone) groups. A two-sided p-value < 0.05 was considered indicative of a statistically significant difference.

Secondary endpoints included:

- change in DAI from Day 4 (treatment initiation) to Day 11, comparing the combined treatment group with control and monotherapy groups;
- changes in mean body weight over the treatment period as a measure of systemic disease burden; and
- histopathological severity scores of distal colon sections assessed at Day 11.

### Data analysis and statistical procedures

This study was designed as an exploratory, preclinical proof-of-concept experiment. No formal a priori power calculation was performed; group size (*n* = 6 per treatment) was selected based on ethical considerations and common practice in DSS-induced colitis models. All statistical analyses were therefore interpreted descriptively, with P-values considered supportive rather than confirmatory.

Colon length measured at Day 11 was pre-specified as the primary endpoint. Disease Activity Index (DAI), body weight, and histopathological scores were analyzed as secondary endpoints.

Group differences in colon length were evaluated using a one-way analysis of variance (ANOVA), with treatment group as the independent variable. When the overall ANOVA indicated a statistically notable difference among groups, pairwise comparisons between the combined cannabidiol (CBD) and sodium propionate (Na-P) treatment and each control or monotherapy group were performed using two-sided t-tests. No adjustment for multiple comparisons was applied, consistent with the exploratory nature of the study.

To further characterize the relative performance of the combination treatment compared with single-agent treatments, Hsu’s Multiple Comparisons with the Best (MCB) procedure was applied as a supportive exploratory analysis. This approach was used to assess whether the combination treatment demonstrated superiority over the monotherapy groups, without implying confirmatory evidence of synergy.

Disease Activity Index (DAI) data were analyzed using two complementary approaches to assess both longitudinal treatment effects and overall change in disease severity.

Longitudinal DAI measurements collected from Day 4 (treatment initiation) through Day 11 were analyzed using a mixed-effects repeated-measures model. Treatment group was included as a fixed effect, time was specified as the repeated measure, and animal ID was included as a random effect to account for within-subject correlation. An unstructured covariance matrix was used. Least squares means (LSMeans) were derived to summarize adjusted group-level DAI trajectories over time.

In addition, overall change in DAI from Day 4 to Day 11 was calculated for each animal and analyzed using a factorial analysis of variance (ANOVA) model. This model included main effects for CBD and Na-P, as well as their interaction term, with the HA vehicle group serving as the reference condition. This analysis was performed to explore potential additive or interactive effects of the combined treatment.

All DAI analyses were conducted in an exploratory manner and interpreted descriptively.

This was a laboratory study and all animals were closely monitored, therefore no data were missing.

Body weight was recorded daily for each animal throughout the study period. For cross-sectional comparisons, body weight at Day 6 and Day 11 was analyzed separately. Overall differences between treatment groups were assessed using one-way analysis of variance (ANOVA). When applicable, planned pairwise comparisons were used to compare treatment groups with control groups in an exploratory manner.

Histopathological scores were analyzed using one-way ANOVA. Given the ordinal and semi-quantitative nature of the scoring system, these analyses were interpreted descriptively.

A two-sided P-value < 0.05 was considered indicative of a statistically notable difference. All analyses were performed using JMP^®^ Pro version 18.1.0.

## Results

### Colon length

Colon length was assessed on Day 11 as a pre-specified primary endpoint and an established macroscopic marker of disease severity in DSS-induced colitis (Peyrin-Biroulet et al. 2015).

**Fig. 2:**
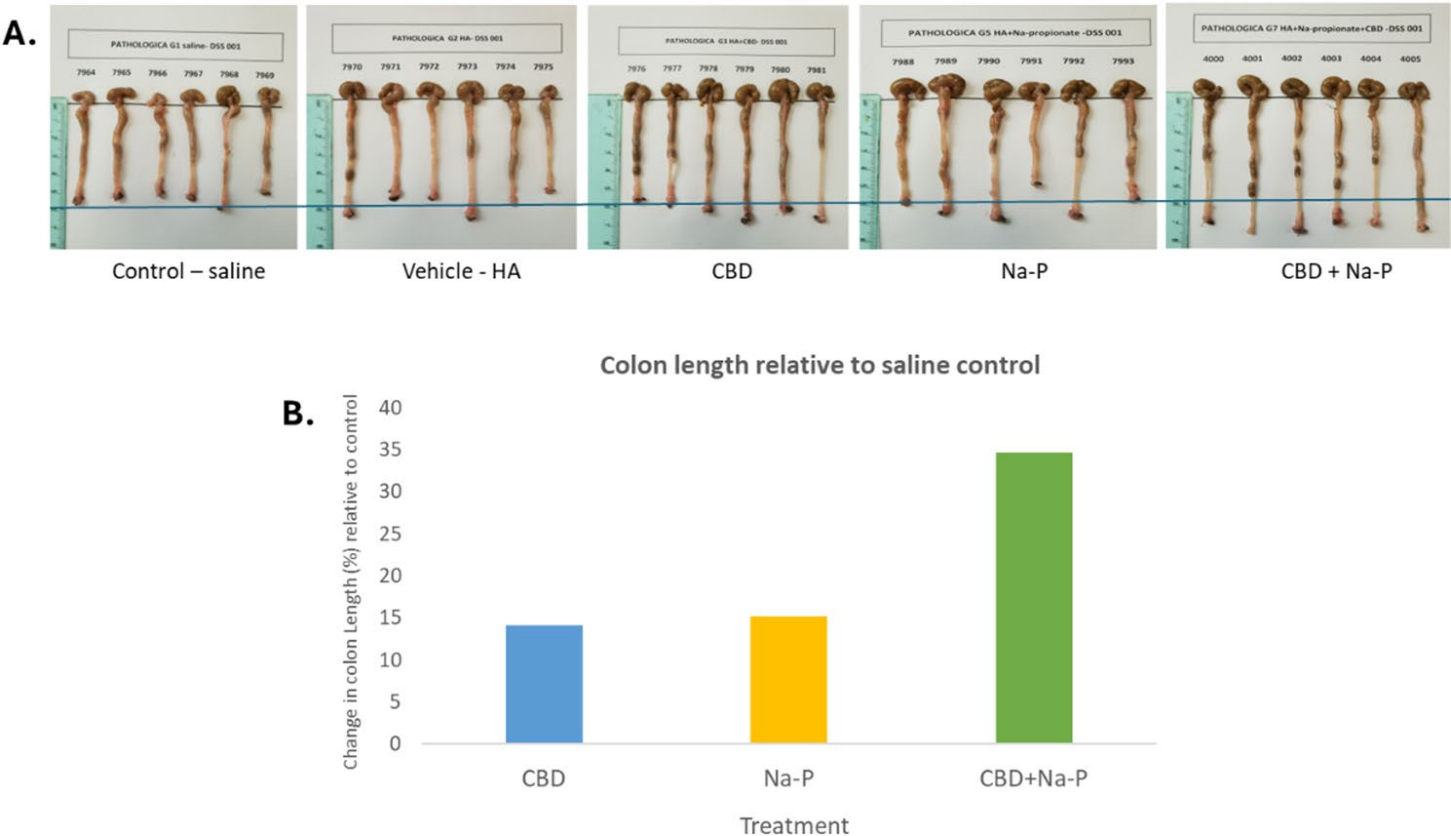
Colon length measured on Day 11. **A** Representative images of excised colons. **B** Quantitative analysis expressed as percentage change relative to saline control. The CBD+Na-P group demonstrated the greatest preservation of colon length. Statistical analysis was performed using one-way ANOVA

DSS-treated control animals exhibited marked colon shortening. Mean colon length in the saline group was 52.83 ± 1.28 cm, and in the HA vehicle group 56 ± 2.11 cm, confirming successful induction of colitis (Fig. 2A).Treatment with either CBD or sodium propionate alone resulted in modest but measurable preservation of colon length. Mean colon length in the CBD group was 60.3 ± 1.39 cm and in the Na-P group 60.83 ± 2.44 cm, corresponding to a 14.2% and 15.1% increase relative to saline controls respectively.

In contrast, animals receiving the combined CBD and Na-P treatment exhibited the greatest preservation of colon morphology, with a mean colon length of 71.17 ± 0.89 cm, corresponding to a 34.7% increase compared with saline controls (*p* < 0.05).

Direct comparisons demonstrated that colon length in the combination treatment group was significantly greater than in either monotherapy group (*p* < 0.05 for both comparisons), indicating enhanced efficacy of the combined intervention.

**Fig. 3:**
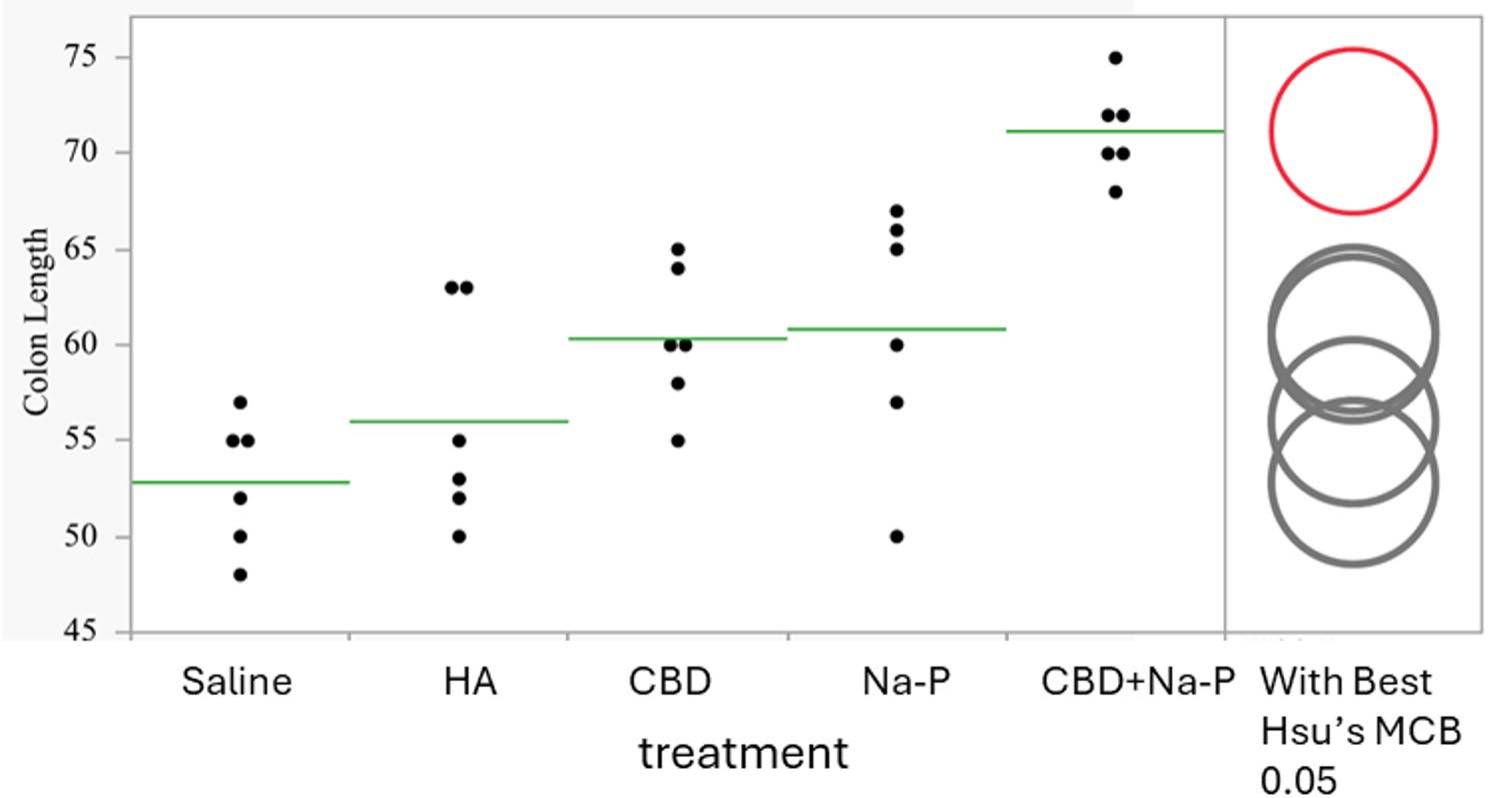
Distribution of individual colon length measurements by treatment group. Each dot represents one animal; horizontal lines indicate group means. Circles on the right denote results of Hsu’s Multiple Comparisons with the Best (MCB) analysis comparing the combination treatment with monotherapies (exploratory analysis). The circles on the right side of chart mark the statistically significant difference of the combined effect over those of the two single treatment effects

Distribution of individual colon length measurements is shown in Fig. 3. Supportive analysis using Hsu’s Multiple Comparisons with the Best (MCB) procedure identified the combination treatment as superior to both single-agent treatments (*p* < 0.001), further reinforcing the robustness and synergy of this effect.

### Disease activity index (DAI)

Following DSS exposure, DAI increased in all groups beginning on Day 3, with comparable scores across groups at Day 4 prior to treatment initiation, indicating a uniform baseline disease severity. Mean DAI values at Day 4 ranged from 1.0 to 1.39 across treatment groups.

**Fig. 4:**
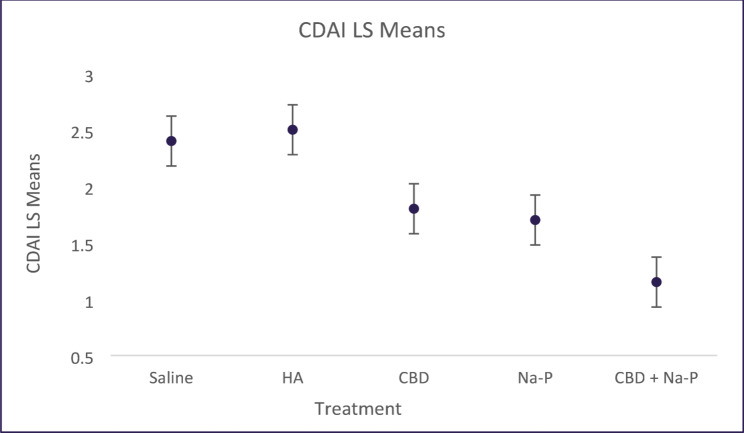
Longitudinal Disease Activity Index (DAI) from Day 4 to Day 11 analyzed using a mixed-effects repeated-measures model. Data are presented as least squares means. Error bars represent ± 1 standard error of the mean (SEM), reflecting the precision of the LSMean estimates. Lower values indicate improved clinical status. The CBD + Na-P group shows numerically lower DAI values over time; overall between-group differences were not statistically significant

Longitudinal analysis using a mixed-effects repeated-measures model demonstrated numerically lower adjusted LSMean DAI values over time in animals receiving the combined CBD and Na-P treatment compared with control and monotherapy groups (Fig. 4). LSMean DAI values across the treatment period were 2.31 for saline, 2.38 for HA, 1.67 for CBD, 1.67 for Na-P, and 1.16 for CBD + Na-P. Overall between-group differences across the full treatment period did not reach statistical significance.

**Fig. 5:**
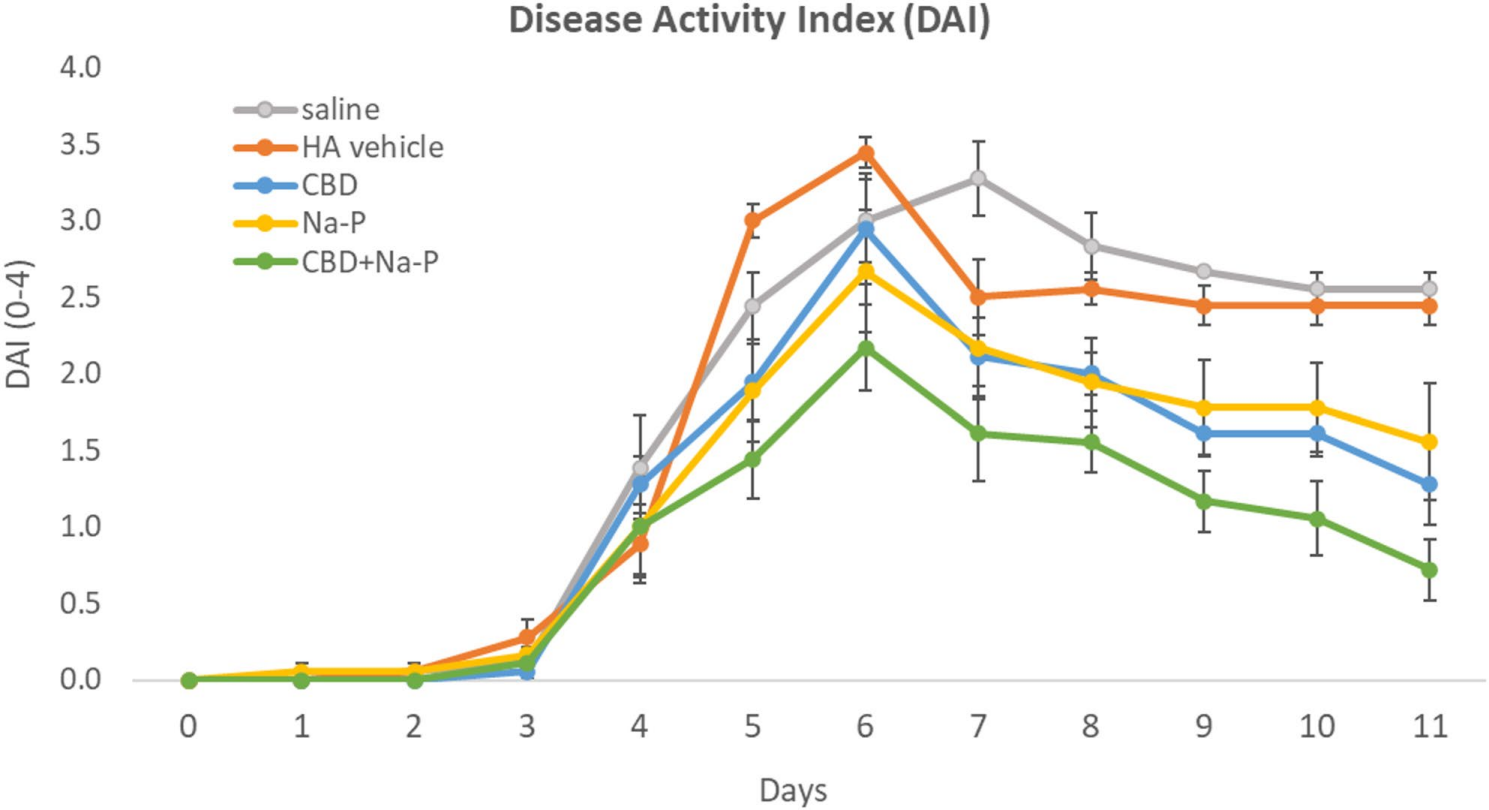
Disease Activity Index (DAI) at individual time points following DSS induction. Data are shown as mean ± SEM. Peak disease severity occurred around Day 6. The CBD + Na-P group exhibited lower DAI values compared with control groups at peak disease and Day 11

Peak disease severity occurred around Day 6, consistent with the DSS colitis model (Chunhua and Didier 2024). At this time point, mean DAI scores were highest in the saline (3.0 ± 0.27) and HA (3.4 ± 0.1) groups. Animals treated with the combined CBD and Na-P enema exhibited significantly lower peak DAI scores (2.17 ± 0.28) compared with both control groups (*p* = 0.0058). Reductions observed in the CBD (2.94 ± 0.36) and Na-P (2.67 ± 0.4) monotherapy groups did not reach statistical significance at this time point (Fig. 5).

To further explore treatment effects, change in DAI from Day 4 to Day 11 was analyzed using an exploratory factorial model. The combination treatment resulted in a numerically greater reduction in DAI compared with either monotherapy. Comparison of Na-P alone versus the combination showed a mean difference of − 0.835 (*p* = 0.052), while comparison of CBD alone versus the combination showed a mean difference of − 0.558 (*p* = 0.183).

Collectively, these findings indicate that while DAI improvements did not consistently reach statistical significance across analyses, the combination treatment demonstrated a reproducible trend toward greater clinical improvement relative to monotherapy and control groups.

### Body weight

**Fig. 6:**
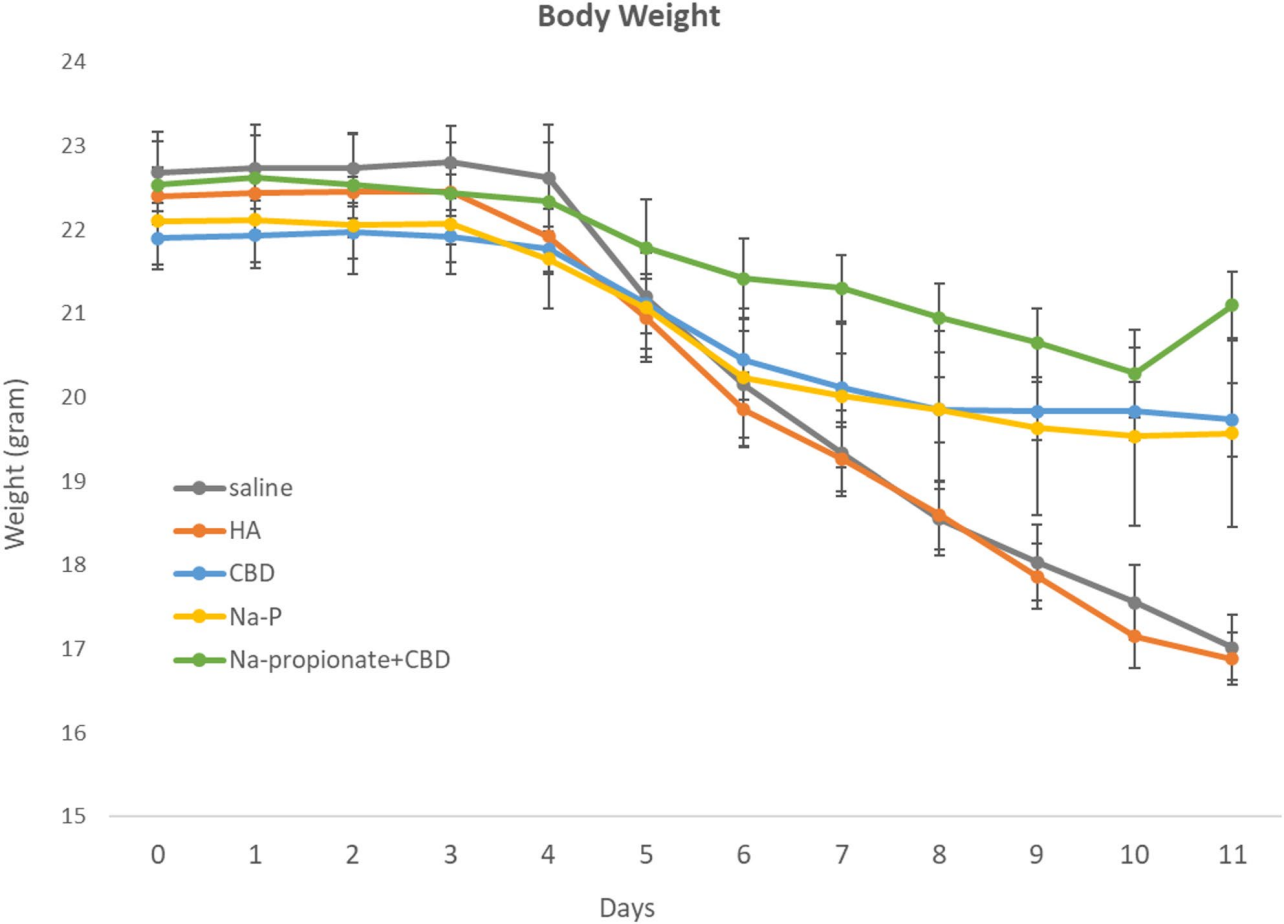
Mean body weight (g) during DSS-induced colitis. Data are presented as mean ± SEM. All groups exhibited weight loss following DSS exposure. Animals treated with the combination of CBD and Na-P demonstrated attenuation of weight loss beginning on Day 6 compared with control groups

**Fig. 7:**
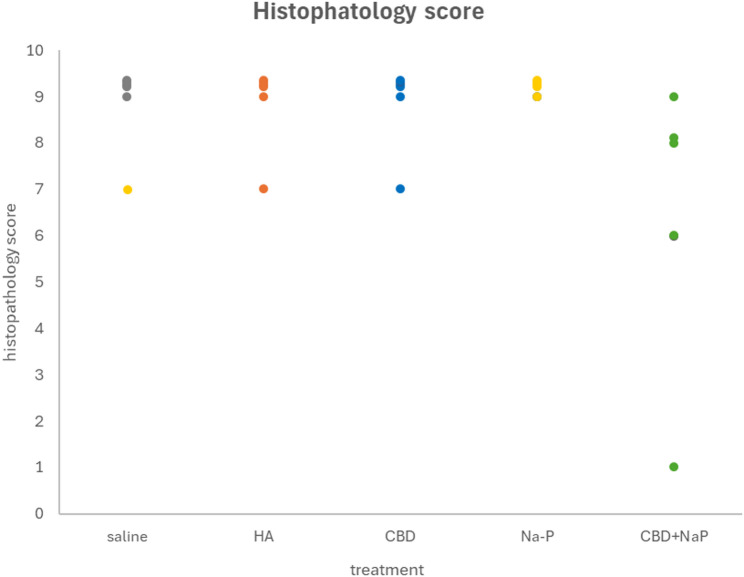
Semi-quantitative histopathological scores of distal colon sections on Day 11. Data are presented as mean ± SEM. The CBD + Na-P group showed numerically lower scores; differences did not reach statistical significance

**Fig. 8:**
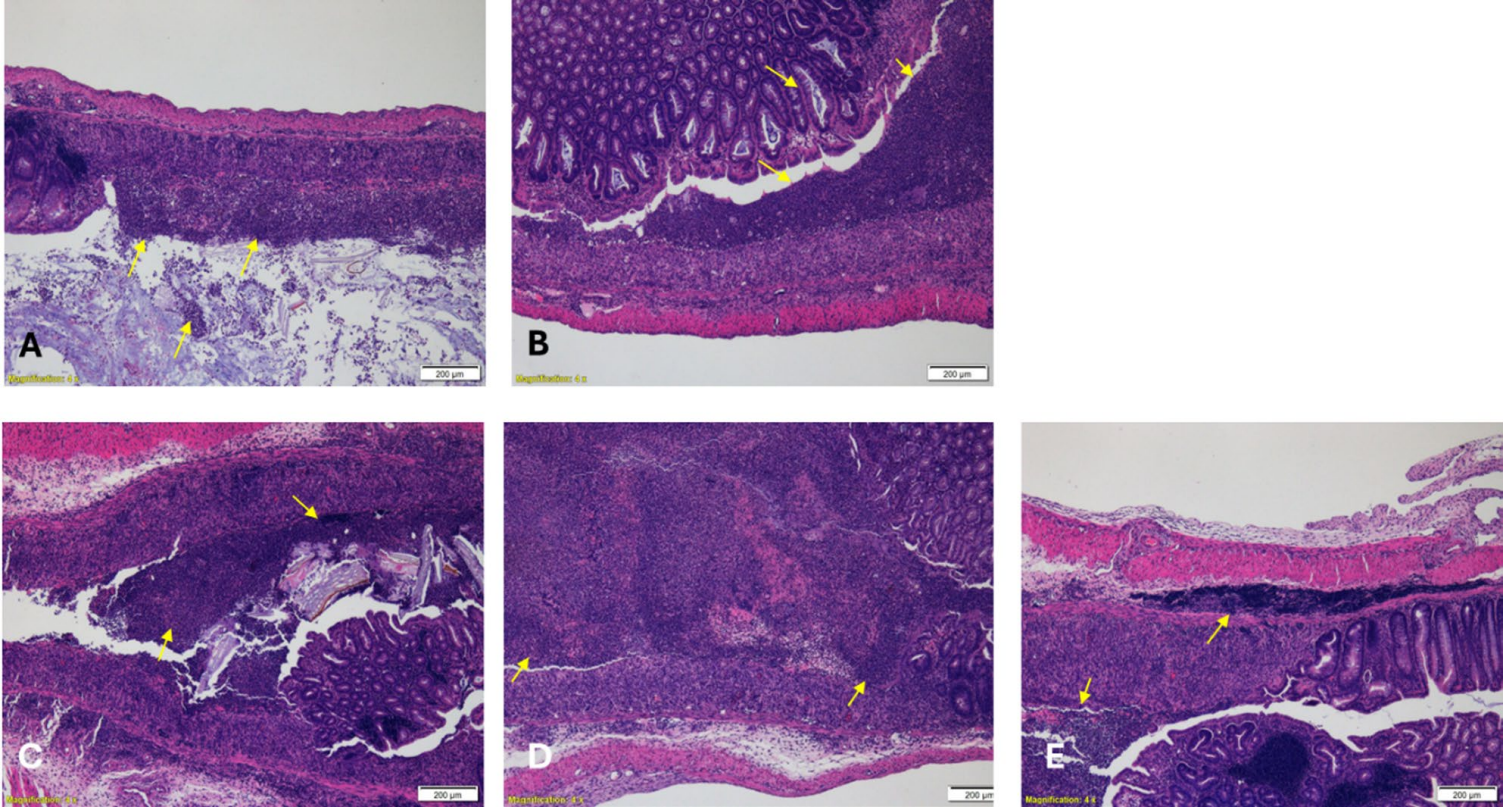
Representative histopathological micrographs of distal colon sections from DSS-induced colitis mice. Hematoxylin and eosin (H&E)–stained sections illustrating colonic mucosal architecture and inflammatory changes across treatment groups: **A** Saline control, Severe ulceration of large parts of the mucosa, erosion and crypt loss with many cellular debris in the gut lumen. Inflammatory reaction around and within the lesions is shown (arrows); **B** Hyaluronic acid (HA) vehicle control, Severe erosion of large parts of the mucosa. Inflammatory reaction within the lesions is shown (arrows); **C** Cannabidiol (CBD), Severe ulceration of large parts of the mucosa, partial erosion and crypt loss. Note inflammatory reaction around and within the lesions (arrows); **D** Sodium propionate (Na-P), Severe ulceration of large parts of the mucosa, partial erosion and crypt loss. Note inflammatory reaction within the lesions (arrows); **E** CBD + Na-P combination therapy, Moderate ulceration of the mucosa, erosion and crypt loss. Inflammatory reaction around and within the lesions is shown (arrows). Images are representative of blinded histopathological evaluations and correspond to semi-quantitative scoring data. Original magnification ×4

Body weight was monitored daily throughout the study period, and group-specific trajectories are shown in fig. 6. At Day 6, mean body weight did not differ significantly among treatment groups. Mean body weight at Day 6 was 20.15 ± 1.71 g and 19.85 ± 1.18 g in the saline and the HA groups respectively. Animals treated with CBD or Na-P alone had comparable mean body weights (20.45 ± 1.30 g and 20.23 ± 2.22 g, respectively), while the combined treatment group showed a numerically higher mean body weight (21.42 ± 1.28 g). One-way ANOVA revealed no statistically significant differences between groups at this time point (*p* = 0.51) (Figs. 7 and 8).

By Day 11, significant differences in body weight were observed across treatment groups. Mean body weight in the saline and HA control groups was 17.02 ± 1.05 g and 16.88 ± 0.84 g, respectively. In contrast, animals treated with CBD (19.73 ± 1.18 g) or Na-P (19.57 ± 2.97 g) exhibited higher body weights compared with controls. Animals receiving the combined CBD + Na-P treatment demonstrated the greatest preservation of body weight, with a mean value of 21.10 ± 1.05 g. One-way ANOVA confirmed a significant overall treatment effect at Day 11 (*p* = 0.0003).

### Histopathological findings

Overall, significant histopathological lesions were observed across the groups, characterized by extensive mucosal ulceration, epithelial necrosis, and inflammatory cell infiltration. The lesions were prominently defined by the loss and necrosis of the mucosal epithelium, accompanied by a robust inflammatory response. In severe cases, a higher presence of neutrophils was noted, further indicating the intensity of the inflammatory process.

The severity of histopathological damage in the different treatment groups, as assessed by semi-quantitative analysis, is summarized below (Fig. 7):

Control groups (Saline and HA) exhibited severe lesions, with an average histopathological score of 8.67 ± 0.33. Extensive mucosal ulceration, erosion, and crypt loss were evident, with large amounts of cellular debris in the intestinal lumen. Inflammatory reactions were prominent, both within and surrounding the lesions.

Both CBD and Na-P monotherapy groups demonstrated histopathological damage similar to that observed in the control groups, with no significant improvement in lesion severity (8.67 ± 0.33 and 8.67 ± 0.33 respectively). Mice receiving the combined treatment exhibited a reduction in histopathological scores (6.33 ± 1.17). Although this reduction was not statistically significant when compared to either compound alone, it approached significance when compared to the HA control group (*p* = 0.06).

In addition, the combination treatment group displayed a lower percentage of neutrophils (~ 21%) compared to the control, CBD alone, and Na-P alone groups (~ 26%), suggesting a potential attenuation of acute inflammation.

No hemorrhages or fibrin deposits were observed in the ulcerated regions across any of the groups.

To support the semi-quantitative histopathological scoring and enable independent visual assessment, representative hematoxylin and eosin (H&E)–stained micrographs from each treatment group were included (Fig. 8). All images were selected from blinded evaluations and illustrate histological features corresponding to the reported scores, including epithelial integrity, inflammatory cell infiltration, crypt architecture, and mucosal ulceration. These representative sections corroborate the scoring data, demonstrating reduced mucosal damage, improved crypt preservation, and attenuated inflammatory infiltration in the combined treatment group compared with control and monotherapy groups.

## Discussion and conclusions

In this study, we demonstrated the efficacy of a novel enema formulation combining CBD and Na-P in attenuating the clinical, morphological, and histological manifestations of DSS-induced colitis in mice. While CBD and Na-P have previously been investigated as individual therapeutic agents, our findings highlight their complementary actions and suggest potential synergistic benefits when administered together.

Our primary endpoint, colon length, showed the most pronounced improvement in the combined therapy group, with significantly longer colons compared with control and monotherapy treated animals. Moreover, the significant differences observed between the combined therapy and monotherapy groups provide additional support for a synergistic benefit of combined CBD and Na-P treatment. This difference was further emphasized in post hoc and more robust statistical analysis such as Hsu test.

Assessment of the Disease Activity Index (DAI) showed that although monotherapy treatment with CBD or Na-P alone reduced disease severity compared with controls, the combination therapy produced a steeper and more sustained decline in DAI scores over time. These findings suggest that simultaneous targeting of pro-inflammatory pathways by CBD and enhancement of mucosal barrier integrity through SCFA-mediated mechanisms by Na-P may result in a more robust therapeutic effect. Notably, the combination treatment showed statistically significant improvement of DAI scores compared to control groups and a trend of improvement compared to monotherapy, mostly evident at Day 11 and demonstrating a consistently superior trend throughout the treatment period.

Analysis of individual DAI components further underscored the protective effect of the combination therapy, particularly with respect to body weight preservation. Mice receiving the combined treatment exhibited significantly attenuated weight loss compared with controls, with differences becoming apparent from Day 6 onward.

Histopathological evaluation further confirmed the beneficial effects of the combination therapy, revealing reduced mucosal ulceration, epithelial necrosis, and inflammatory cell infiltration relative to controls and monotherapy. Although reductions in overall histopathological scores did not reach statistical significance when compared with monotherapy, they approached significance relative to the saline and HA vehicle control. Importantly, the combination group exhibited a lower proportion of neutrophil infiltration compared with both control and monotherapy groups, suggesting a potential mechanism by which the combined treatment mitigates acute inflammatory responses.

No hemorrhages or fibrin deposition were observed in any treatment group, indicating that neither CBD nor Na-P exacerbated these pathological features and supporting the favorable safety profile of the combination therapy.

Overall, across all evaluated clinical, macroscopic, and histological parameters, the combination treatment consistently outperformed both controls and monotherapies. These findings further emphasize the potential advantage of a combined CBD and Na-P approach over single-agent therapy in the management of ulcerative colitis.

The complementary mechanisms of action of CBD and Na-P may explain the observed positive effects. CBD’s role in suppressing pro-inflammatory cytokines, such as TNF-α and IL-6, and promoting regulatory T-cell activity addresses key drivers of immune-mediated inflammation(Malfait et al. 2000). Meanwhile, Na-P’s activation of G-protein coupled receptors (GPR41 and GPR43) on epithelial cells and immune cells helps reinforce the intestinal barrier and further modulate inflammation (Tan et al. 2014; Kim and Kim 2017a, b). The combined targeting of immune regulation, epithelial repair and barrier function likely underpins the enhanced efficacy of the combination observed in this study.

The findings of the present study align with previous literature describing the anti-inflammatory and mucosal-protective properties of both agents. Borrelli et al. (Borrelli et al. 2009) and Irving et al. (Irving et al. 2018; Naftali et al. 2018) demonstrated that CBD administration in DSS-induced and clinical UC models decreased cytokine production and improved histological damage. However, oral CBD has limited colonic bioavailability, which likely explains inconsistent clinical efficacy. Our rectal administration approach directly delivers the compound to the inflamed mucosa, paralleling the strategy used by Naftali et al. (Naftali et al. 2024) to achieve symptomatic improvement in UC patients.

Similarly, Na-P, like other short-chain fatty acids, has been shown to promote epithelial regeneration and suppress pro-inflammatory signaling through GPR43 and HDAC inhibition (Tan et al. 2014; Kim and Kim 2017a, b). Propionate also enhances the differentiation of colonic regulatory T cells (Smith et al. 2013), reinforcing immune tolerance. These effects are consistent with our findings of reduced disease activity and improved colon morphology. The superiority of the combination over monotherapies in our study supports a mechanistic complementarity between the two agents: CBD primarily downregulates cytokine-driven inflammation, while Na-P strengthens epithelial barrier integrity and regulates local immune responses.

This dual mechanism is further supported by evidence that both CBD and SCFAs can enhance tight junction proteins such as occludin and claudin-1, improving mucosal integrity and permeability (Smith et al. 2013). The synergy observed in our study likely results from simultaneous modulation of immune signaling and epithelial metabolism - two key axes in UC pathogenesis.

With respect to safety, rectal administration of the combined CBD and Na-P enema was well tolerated throughout the study period. No treatment-related adverse events were observed in animals receiving the combination therapy compared with control or monotherapy groups. Importantly, histopathological evaluation did not reveal evidence of hemorrhage or fibrin deposition in any treatment group, indicating that the combination therapy did not exacerbate mucosal injury or impair tissue integrity. In addition, no signs of increased distress, abnormal behavior, or premature mortality were observed in treated animals. Collectively, these findings suggest a favorable local safety profile for the combined CBD and Na-P enema in this preclinical model.

Although this study demonstrates promising preclinical evidence of the efficacy of CBD and Na-P combination therapy there are some limitations. While the DSS-induced colitis model closely mimics human UC, it is not an identical model, and translating these findings to clinical settings will require further validation through clinical trials.While our findings are promising, the study did not include the molecular or immunohistochemical analyses necessary for mechanistic confirmation, such as the evaluation of cytokine levels or epithelial barrier markers. Furthermore, questions regarding optimal dosing and treatment duration persist. To build on these results, future investigations should explore the pharmacokinetics of rectal CBD/Na-P delivery, analyze microbiome alterations, and evaluate the role of maintenance therapy in long-term mucosal healing. The standard treatment for mild ulcerative proctitis begins with aminosalicylates, which are generally safe, cost-effective, and well-tolerated. However, when this treatment proves insufficient, patients are often escalated to therapies such as corticosteroids, immunosuppressants, and biologic drugs. These treatments, typically reserved for severe cases, carry significant side effects and financial burden. Unfortunately, there is a significant gap in the management of proctitis—if aminosalicylates fail, topical treatment has to be replaced by systemic treatment with more potent drugs that come with high costs and potential side effects, which may not always be justified. Addressing this unmet need is crucial for providing effective, safer treatment options for moderate proctitis. The A combination of CBD and Na-P may fill this gap, offering a safer, targeted option for controlling inflammation and promoting mucosal healing in moderate cases.

From a translational standpoint, our results complement current clinical goals emphasizing “treat-to-target” mucosal healing in UC (Peyrin-Biroulet et al. 2015). Locally acting, non-systemic therapies such as this enema formulation could provide significant benefit for distal colitis, reducing reliance on systemic immunosuppression. The favorable safety profile observed—absence of hemorrhages, fibrin, or systemic toxicity—supports further development of this approach in early-phase clinical studies. Subsequent studies are warranted to further validate this treatment in human clinical trials.

## Data Availability

The datasets generated and/or analyzed during the current study are available from the corresponding author on reasonable request.

## Abbreviations

UC: Ulcerative colitis
IBD: Inflammatory bowel disease
CBD: Cannabidiol
Na-P: Sodium propionate
DSS: Dextran sodium sulfate
HA: Hyaluronic acid
DAI: Disease activity index
SCFA: Short-chain fatty acid
MCB: Multiple comparisons with the best
MMRM: Mixed model repeated measures
H&E: Hematoxylin and Eosin
SEM: Standard error of the mean
